# Fending for Thyself: Honey Bees From Ethiopia Inflict Physical Damage on *Varroa destructor*

**DOI:** 10.1002/ece3.72660

**Published:** 2025-12-16

**Authors:** Walellign W. Wanore, Christian W. W. Pirk, Abdullahi A. Yusuf, Workneh Ayalew, Beatrice T. Nganso

**Affiliations:** ^1^ International Center of Insect Physiology and Ecology Nairobi Kenya; ^2^ Department of Zoology and Entomology University of Pretoria Pretoria South Africa

**Keywords:** Amhara region, Ethiopia, grooming and hygienic behaviors, *Varroa* mite loads

## Abstract

The ectoparasitic mite *Varroa destructor* and its associated viruses threaten the health of honey bee ( *Apis mellifera* L.). Yet, African populations survive mite infestations without miticide treatment largely due to social immunity behaviors. However, little is known about these defense mechanisms in *A. m. simensis* populations from the Amhara region of Ethiopia. Therefore, this study investigated grooming and hygienic behaviors across lowland, midland, and highland areas during the wet and dry seasons in this region. Grooming behavior was quantified by measuring adult mite infestation rates, daily natural mite fall, and the proportion of mites showing physical damage per colony. Hygienic behavior was evaluated via the removal of pin‐killed brood cells. Results were compared with Kenya's resistant *A. m. scutellata* and susceptible European hybrids in the USA. Honey bees in the Amhara region maintained low mite infestations (< 3.5 mites/100 honey bees) and exhibited higher grooming rates, ranging between 15% to 43% and inflicted more frequently damage to legs and gnathosoma comparable to Kenya's resistant honey bees. In addition to the 10 previously known damage categories inflicted on the mites by honey bees, two new mite damage combinations were identified. Their hygienic behavior was also high, ranging between 79.9% to 98.6% within 24 h and reached 98.8% to 100% after 48 h. While adult grooming and hygienic behaviors significantly varied with landscape and/or season, neither significantly correlated with mite infestation loads, suggesting these traits confer tolerance rather than resistance. Other resistance mechanisms, such as suppressed mite reproduction in worker brood cells, may further reduce colony mite infestations and should be evaluated in future studies.

## Graphical abstract

This study assessed grooming and hygienic behaviors of *Apis mellifera simensis* in Ethiopia's Amhara region across landscapes and seasons. Despite *Varroa* mite levels occasionally exceeding economic thresholds, bees maintained low infestations through strong social immunity traits, suggesting natural tolerance rather than resistance. These findings support sustainable, chemical‐free beekeeping practices in the region.

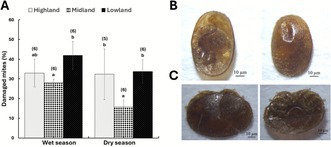

## 1 Introduction

Honey bee ( *Apis mellifera* L.) populations in North America, across Europe, and the Middle East experience considerable summer, winter, and/or annual colony mortality rates (Soroker et al. 2011; Lee et al. 2015; Bruckner et al. 2020; Gray et al. 2023; Nearman et al. 2025). These colony mortality rates threaten both crop pollination and livelihoods dependent on the sale of beehive products such as honey, beeswax, propolis, bee pollen, and venom, among others (Soroker et al. 2011; Lee et al. 2015; Bruckner et al. 2020; Gray et al. 2023; Nearman et al. 2025). Multiple factors contribute to colony death, including pesticide exposure, poor nutrition due to habitat loss, pests, pathogens, and climate change (Reviewed in Hristov et al. 2020). Among these factors, the ecto‐parasitic mite *Varroa destructor* and the viruses it transmits remain the primary drivers of colony mortality globally (Hristov et al. 2020; Traynor et al. 2020).

*Varroa* mite is an ecto‐parasite of honey bees, commonly found on both developing and adult honey bees inside the hive (Francis et al. 2013). The mites feed primarily on the fat body but also on the hemolymph of their honey bee host (Ramsey et al. 2019), compromising their immune defenses and increasing susceptibility to secondary infections, particularly the viruses transmitted by the mites (Aronstein et al. 2012; Grozinger and Flenniken 2019). The combined effects of *Varroa* parasitism and viral pathogenicity are now considered the leading cause of colony mortality in the affected countries (Hristov et al. 2020; Traynor et al. 2020). European honey bee populations are particularly vulnerable to *Varroa* infestations due to limited expression of adaptive behaviors seen in the mite's original host, the eastern honey bee *A. cerana* in Southeast Asia (Rosenkranz et al. 2010). These behaviors include entombing of mite‐infested drone brood, grooming behavior (honey bees' ability to inflict physical damage on the mites upon removal from their bodies or those of their nestmates), hygienic behavior (nursing honey bees' capacity to identify, uncap, and/or remove parasitized brood) (Rosenkranz et al. 2010), and restriction of mites' reproduction to drone cells, which are less common and seasonal (Rath 1999; Rosenkranz et al. 2010). In addition to limited expression of these behavioral traits in *A. mellifera* , *Varroa* mite reproduces in both worker and drone brood cells, and this is not the case in *A. cerana* colonies (Rath 1999). Beekeepers therefore rely on miticides or selective breeding for phenotypic traits to curb colony death attributed to *Varroa* mite and its associated viruses (Rosenkranz et al. 2010; Spivak and Danka 2021; O'Connell et al. 2025).

Interestingly, some *A. mellifera* populations in parts of Africa, Europe, North and South America survive *Varroa* infestations without miticide treatment (Locke 2016). Notably, African honey bee populations in Kenya (Nganso et al. 2017, 2018; Cheruiyot et al. 2018), Tunisia (Boecking and Ritter 1993), and South Africa (Strauss et al. 2013, 2016) exhibit various behavioral traits to combat *Varroa* mite infestations. These traits include *Varroa‐*specific hygienic behavior (the workers' ability to detect and eliminate broods infested with *Varroa*) (Cheruiyot et al. 2018), grooming behavior (Boecking and Ritter 1993; Nganso et al. 2017), suppression of mite reproductive success in worker brood cells (Nganso et al. 2018; Strauss et al. 2016), and a high level of cell‐recapping behavior (Martin et al. 2019). The synergistic effects of these traits help maintain low mite levels (Gebremedhn et al. 2019; Nganso et al. 2017) and virus loads (de Souza et al. 2021) in these surviving African colonies. As such, colony mortality due to *Varroa* mite and its associated viruses, as observed in the Northern Hemisphere, is absent in Africa (Pirk et al. 2014; Nganso et al. 2025).

Honey bee populations from Ethiopia belong to the subspecies *A. m. simensis* (Meixner et al. 2011; Hailu et al. 2020; Wanore et al. 2025). However, little is known about their survival mechanisms against *Varroa*, despite reports of promising survival in some populations without requiring any human intervention. For instance, populations from the Tigray region of Ethiopia survive mite infestations through remarkable hygienic behavior and reduced mite reproduction in worker brood cells (Gebremedhn et al. 2019). However, it remains unclear whether honey bee populations in other regions of the country exhibit similar survival strategies against *Varroa* mites. Moreover, the impact of environmental factors such as landscape classes and seasonality on the expression levels of the behavioral traits employed by African honey bees against *Varroa* is still unclear.

In this study, we tested whether colony defense behaviors contribute to *A. m. simensis*'s survival against *Varroa* mite in the Amhara region of Ethiopia. Specifically, we examined grooming and hygienic behaviors of these honey bees against *Varroa*, and how the expression of these behaviors is influenced by landscape and/or season. We also compared mite infestation and grooming levels in this honey bee subspecies with the surviving *A. m. scutellata* from Kenya and the susceptible *A. mellifera* hybrids of European origin from the USA (Nganso et al. 2017).

## 2 Materials and Methods

### 2.1 Study Area

The study was conducted in the Amhara region (11^o^39′38.88″ N and 37^o^57′28.08″ E), situated in the northwestern and north central parts of Ethiopia and covering a total area of approximately 170,000 km^2^, with elevations between 506 to 4517 m above sea level (a.s.l.), and a diverse flora and fauna. The mean annual rainfall ranges between 770 to 2000 mm, while the average annual air temperature is from 16°C in the summer to 27°C in the dry season (Midekisa et al. 2015). Nearly 90% of the region's population resides in rural areas and relies on agriculture for their livelihood. Land use includes 4,679,468 ha of temporary crops, 59,643 ha of permanent crops, 88,852 ha of fallow land, 331,714 ha of grazing land, over 112,535 ha of woodland, and 242,784 ha of other land use types; totaling 5,514,995 ha (Central Statistical Agency (CSA) 2021). Amhara is the second‐largest honey producing region in Ethiopia, where 21% of the colonies and 26% of the annual honey production come from (Dafar and Turi 2019). The region has two honey harvesting seasons: November to December (the primary honey flow) and March to May (the minor honey flow).

**FIGURE 1:**
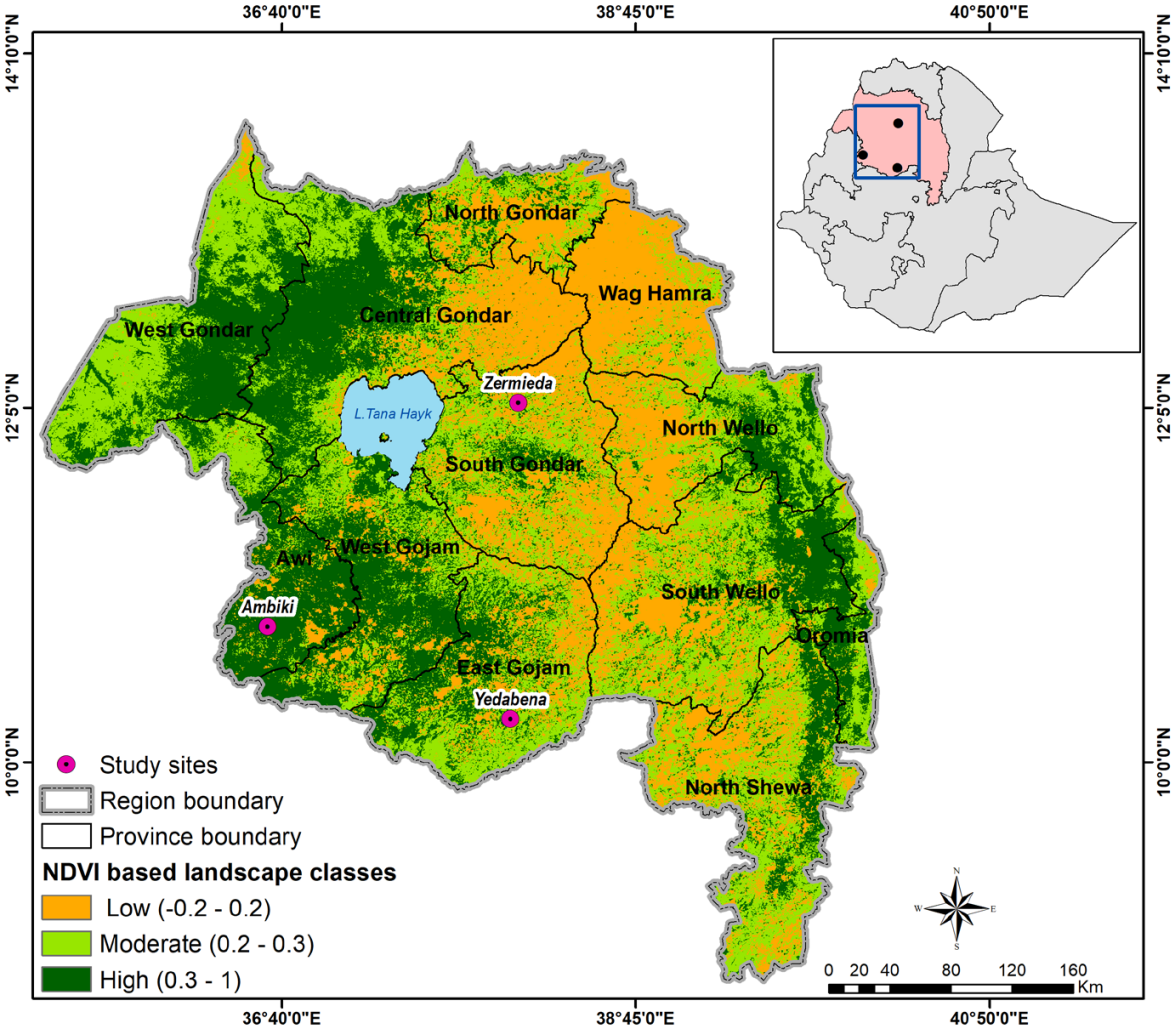
Map of Amhara region in Ethiopia with the location of the three study sites (pink dots). The three levels of the normalized difference vegetation index (NDVI) are indicated by different color pixels. The inset figure provides an overview of the position of the Amhara region and the study sites within Ethiopia.

Three study sites were selected for this study: Ambiki, Yedabena and Zermieda, located in the Guangua, Awabel, and Ebinat districts, respectively (Figure 1). These study sites were located within the highland, midland, and lowland landscape types, respectively, based on the Normalized Difference Vegetation Index (NDVI), which is a proxy for the net primary productivity of ecosystems. The NDVI was obtained by dividing the difference between the near‐infrared (NIR) and red (R) band reflectance by their summation [(NIR−R)/(NIR + *R*)]. We used Google Earth Engine (GEE) platform for processing and analyzing of satellite images obtained from Sentinel 2, with a spatial resolution of 10 m (https://earthengine.google.com). The NDVI thresholds were established to delineate the three landscape classes: lowland, midland, and highland, defined as −0.2–0.2, 0.2–0.3 and 0.3–1, respectively. The average relative humidity, maximum and minimum temperatures during the wet and dry seasons of Awi, East Gojjam, and South Gondar provinces where Ambiki, Yedabena and Zermieda study sites are located, respectively, are shown in Table S1.

From each study site, six queenright colonies, each headed by naturally mated queens housed in Langstroth hives, were selected for the assessment of hygienic and grooming behaviors, as well as mite infestation rates. Data collection occurred during both the wet (November—December 2022) and dry (January—February 2023) seasons (Table S1). Unlike the wet season, the dry season is characterized by reduced brood rearing within honey bee colonies. For the dry season, we also compared adult mite infestation levels and grooming rates of these Ethiopian honey bee populations with previously reported data on the surviving *A. m. scutellata* in Kenya and the susceptible *A. mellifera* hybrids of European origin in the USA (Nganso et al. 2017). Fourteen and 20 queenright colonies were sampled from Kenya and the USA in this previous study, respectively (Nganso et al. 2017).

### 2.2 Determination of Mite Infestation Levels on Adult Workers and Molecular Identification of *Varroa destructor* Mites

The infestation level of *Varroa* mites per 100 adult worker honey bees was determined in each experimental colony during the wet and the dry seasons using the powdered sugar roll method (Dietemann et al. 2013). Thereafter, two mites were collected randomly per colony (from two colonies sampled in each site) for subsequent haplotype identification, targeting the cytochrome oxidase I (Cox1), cytochrome oxidase III (Cox3), and ATP synthase 6 (ATP6) mitochondrial genes and stored in 95% ethanol for DNA analysis Nganso et al. (2017). Totally, six mites were sampled from six colonies. The Isolate II Genomic DNA kit (Bioline, A Meridian Life Science company, Germany) was used for genomic DNA extraction from individual mites following the manufacturer's protocol. Three fragments of mitochondrial genes from cytochrome oxidase I (Cox1), cytochrome oxidase III (Cox3), and ATP synthase 6 (ATP6) were amplified by polymerase chain reaction after measuring the nucleic acid concentration of each sample. Reactions were run in 25 μL volume using MyTaq DNA Polymerase (Meridian Bioscience, USA), under cycling conditions: initial denaturation at 94°C for 4 min, followed by 35 cycles of denaturation at 94°C for 30 s, annealing for 30 s, and extension at 72°C for 1 min. The amplified DNA fragments were separated using 1% agarose gel and purified using the ISOLATE II PCR and Gel Purification Kit (BIOLINE, A Meridian Life Science Company). The obtained PCR products underwent bidirectional Sanger sequencing at Macrogen, Europe. The obtained sequences were then edited with BioEdit (Hall 1999), and consensus sequences were generated using MEGA11 software. DNA sequences from individual mites were analyzed using BLASTn on the National Center for Biotechnology Information (NCBI) platform to identify the *Varroa* mite strain. Species‐level identification was considered reliable when sequence similarity was ≥ 97%. The primer names, sequences, details of the amplicons, and their respective annealing temperatures are provided in Table S2.

### 2.3 Assessment of Honey Bee Grooming Behavior

During both seasons, grooming behavior was assessed in each experimental colony using the standard screen bottom board method, as described before (Bienefeld et al. 1999; Corrêa‐Marques et al. 2000; Dietemann et al. 2013; Nganso et al. 2017). Mite collection from the debris on the bottom board was conducted daily for up to 7 days using a fine camel hairbrush. The collected mites were examined for injuries under a Leica EZ4D microscope (×40 Magnification) with Leica Application Suite v3.4.0 2016 software (Leica Microsystems Limited, Switzerland). Injured mites were grouped into their respective damage categories based on the 10 previously known damage categories—damaged legs (DL), hollow in the dorsal shield (HDS), empty dorsal shield (EDS), damaged shield (DS), damaged shield + damaged legs (DS + DL), hollow in the dorsal shield + damaged legs (HDS + DL), damaged ngathosoma (DG), damaged empty dorsal shield (DEDS), damaged legs + damaged gnathosoma, (DL + DG), and damaged legs + damaged gnathosoma + damaged shield (DL + DG + DS) (Corrêa‐Marques et al. 2000; Nganso et al. 2017). The percentage of damaged mites per colony, considered as the colony's grooming behavior, was calculated by dividing the number of damaged mites by the total number of naturally fallen mites.

### 2.4 Assessment of Honey Bee Hygienic Behavior

The “pin‐killed test”, involving 100 worker cells containing pupae at the purple‐eye stage, was used to determine the hygienic behavior of all experimental colonies only during the wet season, as described in detail by Dietemann et al. (2013). Unfortunately, we were unable to assess hygienic behavior during the dry season due to a considerable shortage of brood inside the colonies. The total number of fully removed pin‐killed brood cells from the test patch was recorded 24 and 48 h thereafter. A colony's hygienic behavior was expressed as the percentage of fully removed pin‐killed brood cells from a test patch of pin‐killed brood cells within 24 and 48 h intervals (Locke and Fries 2011; Nganso et al. 2017).

### 2.5 Statistical Analysis

The data were analyzed using R‐Software version 4.5.1(R Core Team 2023). A Poisson generalized linear model (GLM) with a log link function was employed to compare the *Varroa* mite infestation level/100 adult worker honey bees and daily mite fall/colony across landscape types, seasons, and their interaction. Model significance was assessed via the analysis of deviance with the chi‐squared test when overdispersion was absent, and pairwise comparisons were performed using Tukey's test implemented in the “*emmeans*” package (Lenth 2023). Meanwhile, a Quasi‐Poisson GLM with a log link function was fitted when overdispersion was present, and model significance was analyzed with the *F*‐tests, followed by Tukey's multiple comparisons test for group differentiation.

For the ratio of total natural fallen mites to mite infestation level/100 adult worker honey bees, which represents the portion of mites removed from bees through grooming relative to the total mite population in the colony, normality and homogeneity of variances were first assessed using the Shapiro–Wilk and Levene's tests, respectively, before fitting the model to compare this parameter among landscape types during the wet and dry seasons. These data were log‐transformed to stabilize the variances since all values were positive. When the log‐transformed data met both assumptions during the dry season, a GLM with Gaussian family and the identity link function was fitted to the transformed data, and the analysis of deviance with the *F*‐test was used to assess model significance. When these assumptions were violated during the wet season, a Gamma GLM with a log link was fitted to the untransformed data, and the analysis of deviance with the chi‐squared test was used to assess model significance due to the absence of overdispersion in the data. Pairwise multiple comparison with Tukey's test was performed afterward to identify group differences.

A binomial or quasi‐binomial GLM with a logit link function and binomial distribution error was used to compare the percentage of damaged mites, brood removal at 24 and 48 h intervals across landscape type, season, and their interaction when overdispersion of the data was absent or present, respectively. Model significance was evaluated using the analysis of deviance with the chi‐squared or *F*‐tests, respectively, followed by Tukey's pairwise comparisons implemented in the “emmeans” package.

To compare adult mite infestation levels and daily mite fall/colony among Ethiopian honey bees (this study) and those from Kenya and the USA (data obtained from Nganso et al. 2017) during periods of reduced brood rearing, a Quasi‐Poisson GLM was fitted due to overdispersion of data. Model significance was assessed using the analysis of deviance with the *F*‐tests, followed by Tukey's multiple comparisons test for group differentiation. Adult grooming behavior among these groups was also compared using a Quasi‐binomial GLM with a logit link function and binomial distribution error as described above. Spearman's rank order correlation analysis was employed to test the nature of the relationship between adult mite infestation level, the percentage of damaged mites, hygienic behavior, daily mite fall/colony, the ratio of total natural fallen mites to adult mite infestation, brood removal rate at 24 and 48 h intervals, and the individual mite damage categories.

## 3 Results

### 3.1 Mite Infestation Levels on Adult Worker and Molecular Identification of Mite Haplotype

**FIGURE 2:**
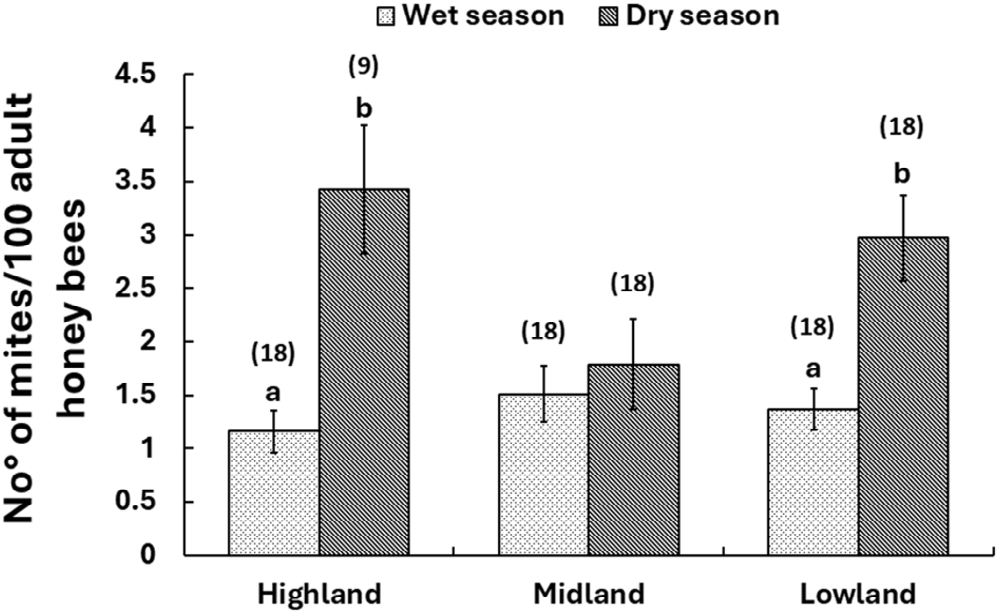
Mite infestation level per 100 adult worker honey bees across sites in wet and dry seasons (Mean ± SEM). Different letters above the bars indicate significant differences between the dry and wet seasons within each apiary site, as determined by the Poisson GLM with log link, *p* < 0.05, followed by post hoc pairwise comparison of means. The numbers above the bars in parentheses represent the total number of replications per site per season (the test was conducted in three replications per colony at a time) (*N*).

We found an effect of season on *Varroa* infestation levels per 100 adult workers (Poisson GLM: *χ* ^2^_(1,95)_ = 84.4, *p* < 0.001), but not of landscape (Poisson GLM: *χ* ^2^_(2,96)_ = 104.5, *p* > 0.05) and the interaction between landscape and season (Poisson GLM: *χ* ^2^_(2,93)_ = 78.4, *p* > 0.05). Specifically, mite infestation levels on adult honey bees were higher during the dry season compared to the wet season, showing a three‐fold increase in Ambiki located in the highland area (Poisson GLM: *χ* ^2^_(1,25)_ = 19.5, *p* < 0.001) and a two‐fold increase in Zermieda located in the lowland area (Poisson GLM: *χ* ^2^_(1,34)_ = 22.3, *p* < 0.001) (Figure 2). However, there was no significant seasonal difference in mite infestation at the Yedabena apiary located in the midland area (Poisson GLM: *χ* ^2^_(1,34)_ = 36.7, *p* > 0.05). The mite species detected across these study sites belong to the Korean strain (K1 haplotype).

### 3.2 Grooming Behavior of Ethiopian Honey Bees Against *Varroa* Mite

**FIGURE 3:**
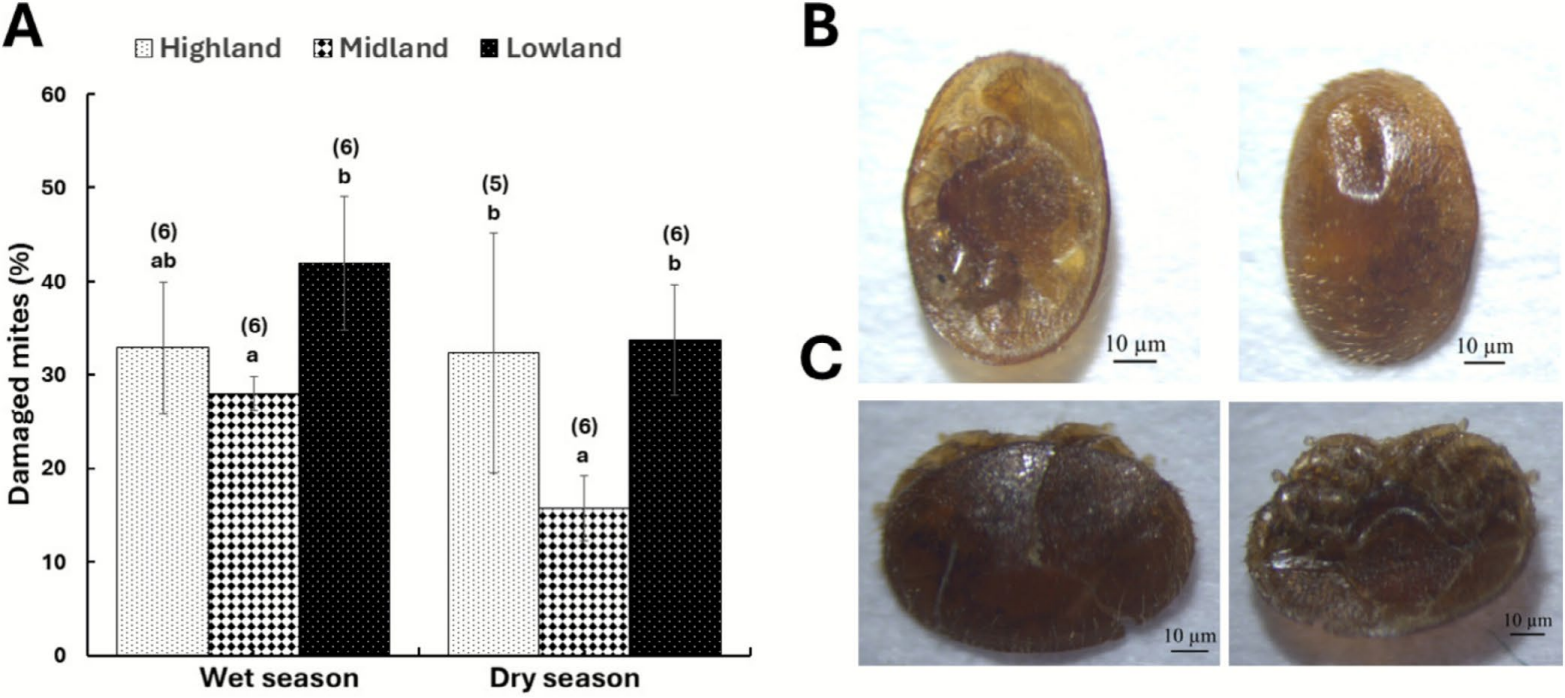
Comparison of grooming behavior of Ethiopian honey bees against mites across different landscape types during the wet and dry seasons (Mean ± SEM) (A). Different letters above bars indicate significant differences among honey bees in the different landscape types when compared using the quasibinomial GLM, followed by post hoc pairwise comparison of means with “landscape” as an explanatory factor, *p* < 0.05. The numbers in brackets above the bars indicate the total number of colonies assessed per season in each landscape type. New combinations of damaged categories: Hollow in the dorsal shield + damaged leg + damaged gnathosoma (B) and damaged gnathosoma + damaged shield (C).

The grooming behavior of honey bees from Ethiopia against the *Varroa* mite was significantly influenced by both landscape (Quasi‐binomial GLM: *F*_(2,31)_ = 13.6, *p* < 0.001) and season (Quasi‐binomial GLM: *F*_(1,30)_ = 5.8, *p* < 0.05), but not by their interaction (Quasi‐binomial GLM: *F*_(2,28)_ = 2.1, *p* > 0.05). Specifically, honey bees in the lowland areas exhibited more grooming behavior against the mite than those in midland areas, regardless of the season (Figure 3A). On the other hand, the grooming behavior of honey bees in highlands was between those of midland and lowland areas during the wet season (Quasi‐binomial GLM: *F*_(2,15)_ = 4.0, *p* < 0.05). However, during the dry season, these highland honey bees had grooming rates that were similar to and different from those of lowland and midland honey bees, respectively (Quasi‐binomial GLM: *F*_(2,13)_ = 12.1, *p* < 0.01) (Figure 3A). There was no significant correlation between adult grooming and mite infestation level during the wet (Spearman's rank correlation: *ρ* = −0.1, *p* > 0.05) and the dry (Spearman's rank correlation: *ρ* = 0.3, *p* > 0.05) seasons.

This study documented different types of mite damage (Table 1) and identified two new combinations of damaged mite categories: hollow in the dorsal shield + damaged leg + damaged gnathosoma (Figure 3B) and damaged gnathosoma + damaged shield (Figure 3C). Among these damaged categories, damaged legs (Quasi‐binomial GLM: *F*_(2,31)_ = 3.7, *p* < 0.05), empty dorsal shield (Binomial GLM: *χ* ^2^_(2,31)_ = 14.1, *p* < 0.05), hollow in the dorsal shield + damaged legs (Binomial GLM: *χ* ^2^_(2,31)_ = 14.1, *p* < 0.05), and damaged legs + damaged gnathosoma (Quasi‐binomial GLM: *F*_(2,31)_ = 6.2, *p* < 0.01) were only significantly influenced by landscape (Table 1). Notably, lowland honey bees exhibited the highest rates of damaged legs, empty dorsal shield, and damaged legs + damaged gnathosoma compared to honey bees in midland and highland areas (Tukey's test, *p* < 0.05). However, for hollow in the dorsal shield + damaged legs, lowland honey bees exhibited the highest rate (~three‐fold more) only during the wet season compared to other landscape types (Tukey's test, *p* < 0.05), but highland honey bees inflicted this damaged type considerably higher during the dry season (more than six‐fold) (Tukey's test, *p* < 0.05) (Table 1). Another damaged category, the damaged shield + damaged gnathosoma, was only significantly influenced by season (Binomial GLM: *χ* ^2^_(1,30)_ = 10.3, *p* < 0.05), with a higher rate recorded during the wet season compared to the dry season (Tukey's test, *p* < 0.05) (Table 1). Two damage categories, hollow in the dorsal shield (HDS) (Quasi‐binomial GLM: *F*_(2,28)_ = 7.3, *p* < 0.01) and damaged gnathosoma (Binomial GLM: *F*_(2,28)_ = 34.3, *p* < 0.05), were significantly influenced by landscape, season, and their interaction. The first damage type was more than six times higher in the highland area during the dry season and more than twice as high in the lowland during the dry season (Tukey's test, *p* < 0.05) (Table 1). The second damage type only differed among the landscape types during the wet season, being lowest in the lowland area compared to both the midland and highland areas (Tukey's test, *p* < 0.05) (Table 1).

**TABLE 1:** Comparison of the percentages (mean ± SEM) for the different categories of damages to *Varroa destructor* recorded in Ethiopian honey bees across landscape, season, and their interaction. *p*‐values highlighted in bold indicate the significant effect of landscape, season, or their interaction when compared using the quasibinomial GLM, *p* < 0.05.

Damage categories	Highland		Midland		Lowland		*p*		
	Dry (%)	Wet (%)	Dry (%)	Wet (%)	Dry (%)	Wet (%)	Landscape	Season	Site: Season
Damaged legs (DL)	1.0 ± 1.0	4.4 ± 1.7	2.9 ± 0.7	5.0 ± 1.8	6.9 ± 2.2	7.3 ± 1.3	**< 0.05**	> 0.05	> 0.05
Hollow in the dorsal shield (HDS)	10.3 ± 6.3	4.1 ± 1.9	1.6 ± 0.6	3.5 ± 1.9	0.8 ± 0.5	10.0 ± 1.6	**< 0.05**	**< 0.05**	**< 0.05**
Empty dorsal shield (EDS)	0.0 ± 0.0	0.0 ± 0.0	0.0 ± 0.0	0.2 ± 0.1	0.3 ± 0.3	1.0 ± 0.5	**< 0.05**	> 0.05	> 0.05
Damaged shields (DS)	1.2 ± 1.2	0.7 ± 0.4	1.1 ± 0.6	2.4 ± 0.8	0.3 ± 0.3	1.4 ± 0.9	> 0.05	> 0.05	> 0.05
Damaged shield + damaged legs (DS + DL)	0.0 ± 0.0	0.0 ± 0.0	0.2 ± 0.2	0.4 ± 0.2	1.0 ± 1.0	0.3 ± 0.2	> 0.05	> 0.05	> 0.05
Hollow in the dorsal shield + damaged legs (HDS + DL)	0.0 ± 0.0	2.2 ± 1.4	0.0 ± 0.0	0.0 ± 0.0	0.6 ± 0.5	0.0 ± 0.0	**< 0.05**	> 0.05	> 0.05
Damaged gnathosoma (DG)	0.0 ± 0.0	3.5 ± 1.9	1.5 ± 0.5	3.6 ± 1.3	0.6 ± 0.6	0.2 ± 0.2	**< 0.05**	**< 0.05**	**< 0.05**
Damaged empty dorsal shield (DEDS)	1.2 ± 1.2	0.0 ± 0.0	0.4 ± 0.4	0.1 ± 0.1	0.6 ± 0.6	0.3 ± 0.2	> 0.05	> 0.05	> 0.05
Damaged legs + damaged gnathosoma (DL + DG)	9.8 ± 8.5	11.2 ± 4.1	4.4 ± 1.8	7.3 ± 1.0	16.2 ± 4.4	16.5 ± 6.7	**< 0.05**	> 0.05	> 0.05
Damaged legs + damaged gnathosoma + damaged shield (DL + DG + DS)	0.0 ± 0.0	7.9 ± 5.2	3.6 ± 2.4	4.4 ± 2.2	6.3 ± 2.0	3.0 ± 1.2	> 0.05	> 0.05	> 0.05
Damaged legs + damaged gnathosoma + hollow in the dorsal shield (DL + DG + HDS)^a^	0.0 ± 0.0	0.4 ± 0.4	0.0 ± 0.0	0.8 ± 0.5	0.8 ± 0.5	0.9 ± 0.6	> 0.05	> 0.05	> 0.05
Damaged shield + damaged gnathosoma (DS + DG)^a^	0.0 ± 0.0	0.7 ± 0.7	0.0 ± 0.0	0.3 ± 0.2	0.0 ± 0.0	0.2 ± 0.2	> 0.05	**< 0.05**	> 0.05

^a^ Represents a new combination of damage categories discovered in this study.

The pattern of mite damage also varied across landscape types and season (Table 1). The most frequently observed damage mite category was damaged leg + damaged gnathosoma in the highland and midland areas during the wet season, whereas this category of damage and HDS were most frequent in the midland and highland areas during the dry season, respectively. In the lowland area, damaged leg + damaged gnathosoma was the most frequent in both dry and wet seasons. Only damaged shield + damaged gnathosoma was found to be positively associated with the adult infestation level during the wet season (Spearman's rank correlation: *ρ* = 0.5, *p* < 0.05).

### 3.3 Daily Mite Fall/Colony and Ratio of Total Natural Fallen Mite to Adult Mite Infestation Levels in Ethiopian Colonies

The daily mite fall per colony was influenced by both landscape (Quasi‐Poisson GLM: *F*_(2,32)_ = 11.9, *p* < 0.001) and season (Quasi‐Poisson GLM: *F*_(1,31)_ = 6.9, *p* < 0.05), but not by the interaction between these parameters (Quasi‐Poisson GLM: *F*_(2,29)_ = 0.3, *p* > 0.05). During the wet season, honey bees in the lowland and midland removed significantly more mites from their colonies daily (three‐ and four‐fold, respectively) than those in the highland (Quasi‐Poisson GLM: *F*_(2,15)_ = 5.4, *p* < 0.05) (Figure 3B). Conversely, during the dry season, honey bees in the midland had higher mite removal rates (two‐ and six‐fold more) than those in both highland and lowland areas, respectively (Quasi‐Poisson GLM: *F*_(2,14)_ = 8.6, *p* < 0.01) (Figure 3B). Daily mite fall/colony and adult infestation level during the wet (Spearman's rank correlation: *ρ* = 0.4, *p* = 0.1 > 0.05) and the dry (Spearman's rank correlation: *ρ* = −0.1, *p* > 0.05) seasons were independent.

**FIGURE 4:**
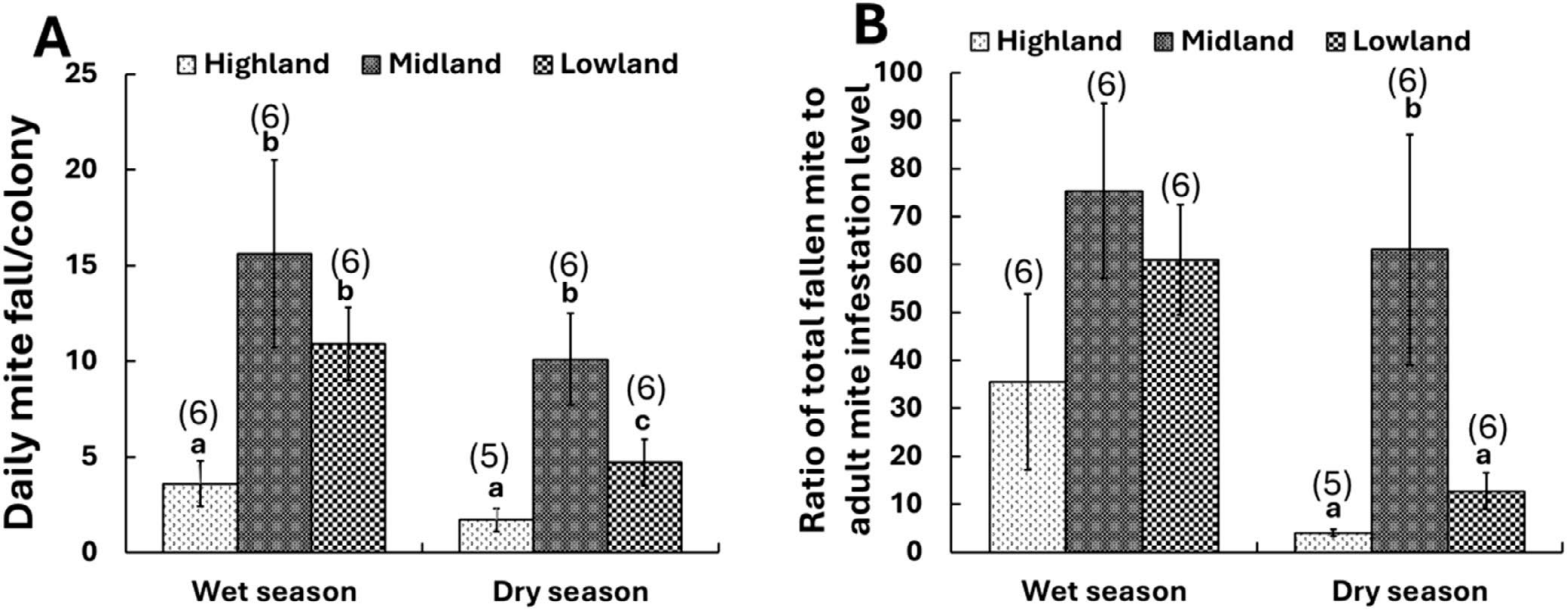
Comparison of the daily mite fall/colony and ratio of total natural fallen mite to *Varroa* infestation level on adult workers among honey bees from Ethiopia found in different landscapes during the wet and dry seasons (Mean ± SEM). Different letters above the bars indicate significant differences among honey bees when compared using the quasi‐poisson GLM, followed by post hoc pairwise comparison of means with “landscape” as an explanatory factor for daily mite fall/colony or the Kruskal–Wallis test, followed by Dunn's post hoc test for the ratio of total fallen mite to adult mite infestation, *p* < 0.05. The numbers in brackets above the bars indicate the total number of colonies assessed per season in each landscape type.

The ratio of total natural fallen mite to *V. destructor* infestation level on adult workers was the same across the landscape types during the wet season (Gamma GLM: *ꭓ* ^2^_(2,15)_ = 14.9, *p* > 0.05) (Figure 4). However, it was five‐ and 16‐fold significantly higher in the midlands than in the low and highlands, respectively, during the dry season (Gaussian GLM: *F*_(2,12)_ = 10.1, *p* < 0.01) (Figure 4). This ratio significantly and negatively correlated with adult mite infestation level during the wet season (Spearman's rank correlation: *ρ* = −0.42, *p* < 0.05) and the dry season (Spearman's rank correlation: *ρ* = −0.53, *p* < 0.05).

### 3.4 Comparison of Adult Mite Infestation Levels and Other Metrics Among Honey Bee Populations From Ethiopia, Kenya and USA During Periods of Reduced Brood Rearing

The adult mite infestation levels differed significantly among honey bees, with *A. mellifera* hybrids of European origin in the USA exhibiting the highest infestation rate (Quasi‐Poisson GLM: *F*_(4,44)_ = 7.5, *p* < 0.001, Table 2). The daily mite fall/colony also varied significantly, with *A. m. scutellata* in Kenya and *A. mellifera* hybrids of European origin in the USA showing the highest daily mite removal rates (Quasi‐Poisson GLM: *F*_(4,46)_ = 3.9, *p* < 0.01). Additionally, the grooming rate against *Varroa* differed among the honey bee subspecies, with *A. m*. *simensis* from the midland area of Ethiopia having relatively the lowest rate, similar to that of Kenyan honey bees (Quasibinomial GLM: *F*_(4,45)_ = 3.5, *p* < 0.05). Interestingly, honey bees from the midland area of Ethiopia showed the highest ratio of total fallen mites to adult mite infestation, which was comparable only to those from Kenya (Gaussian GLM: *F*_(4,44)_ = 7.6, *p* < 0.001).

**TABLE 2:** Comparison of adult mite infestation rates, daily mite fall/colony, percent damaged mites, and ratio of total fallen mites to adult mite infestation rate in colonies of *A. m. simensis* from Ethiopia, *A. m. scutellata* from Kenya, and *A. mellifera* hybrids of European origin from the USA (Mean ± SEM). Different superscript letters (a–d) indicate significant differences between the groups using Tukey's test implemented in the *emmeans* package to identify group differences, *p* < 0.05.

Honey bee subspecies	Adult mite infestation levels	Daily mite fall/colony	Percent damaged mites	Ratio of total fallen mites to adult mite infestation level	References
*A. m. simensis*_H_	3.4 ± 1.2^ab^	1.7 ± 0.6^a^	25.9 ± 11.9^ab^	4.0 ± 0.7^b^	This study
*A. m. simensis*_M_	1.8 ± 0.6^a^	10.1 ± 2.4^b^	15.7 ± 3.5^a^	63.1 ± 24.0^a^	This study
*A. m. simensis*_L_	3.0 ± 0.5^a^	4.7 ± 1.2^c^	33.8 ± 5.9^b^	12.7 ± 3.8^b^	This study
*A. m. scutellata*	5.4 ± 1.4^b^	18.1 ± 2.8^d^	21.3 ± 1.7^a^	52.2 ± 11.3^a^	Nganso et al. (2017)
*A. mellifera* hybrids of European origin	13.8 ± 2.3^c^	15.8 ± 3.9^d^	21.3 ± 2.4^ab^	10.4 ± 2.0^b^	
*p*	**< 0.001**	**< 0.01**	**< 0.05**	**< 0.001**	

*Note:* Subscript letters on *A. m. simensis* “H, M and L” indicate that the honey bees are found in the highland, midland and lowland area, respectively.

### 3.5 Hygienic Behavior of Ethiopian Honey Bees Against *Varroa* Mite

**FIGURE 5:**
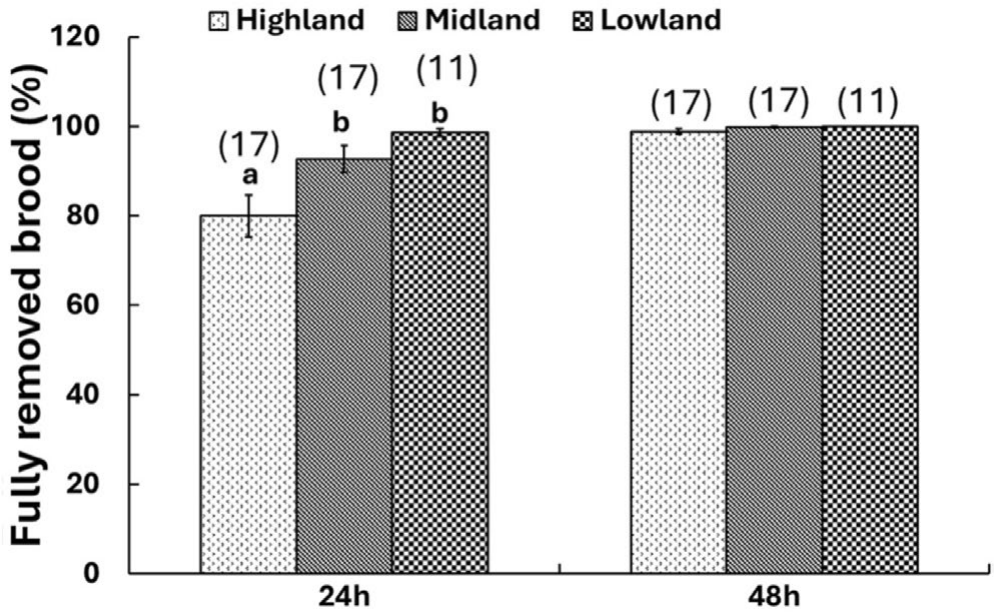
Hygienic behavior of Ethiopian honey bees against mites across different landscapes during the 24 and 48 h interval (Mean ± SE). Different letters above bars indicate significant differences among honey bees in the different landscape types when compared using the quasibinomial GLM, followed by post hoc pairwise comparison of means with “landscape” as an explanatory factor. The numbers in brackets above the bars indicate the total number of replicates assessed during the wet season in each landscape type.

The hygienic behavior of honey bees from Ethiopia was significantly influenced by landscape (Quasi‐binomial GLM: *F*_(2,87)_ = 14.0, *p* < 0.001) and time interval of brood removal (Quasi‐binomial GLM: *F*_(1,86)_ = 50.3, *p* < 0.001), but not by their interaction (Quasi‐binomial GLM: *F*_(2,84)_ = 0.1, *p* > 0.05). This behavior only varied among Ethiopian honey bees within a 24 h interval of pin killing (Quasi‐binomial GLM: *F*_(2,42)_ = 8.0, *p* < 0.01), with those in the highland being relatively less hygienic than those in the lowland and midland—which displayed similar hygienic behavior against the mites (Figure 5). There was no association between hygienic behavior and adult mite infestation level during the 24 h interval (Spearman's rank correlation: *ρ* = −0.1, *p* > 0.05) and the 48 h interval (Spearman's rank correlation: *ρ* = 0.2, *p* > 0.05).

## 4 Discussion

### 4.1 Adult Mite Infestation Level and Molecular Identification of *Varroa* Mite

This study revealed higher adult mite infestation during the dry season when brood rearing declines, leading to greater mite accumulation on adult honey bees compared to the wet season, as *Varroa* mites have fewer brood cells available for reproduction and therefore remain on adult hosts (Rosenkranz et al. 2010). Similar seasonal patterns have been reported from previous research in Western Ethiopia (Begna et al. 2016), Europe (Wilkinson and Smith 2002), and both North (Jack et al. 2023) and South America (Ceccotti et al. 2022). Unlike the lowland and highland honey bees, no significant seasonal variation in adult mite infestation rate was found in honey bee colonies from the midland area, likely due to a consistently high ratio of mite removal relative to the total mite population present in their colonies across seasons (Figure 4B). In contrast to some reports, landscape has no significant effect on mite population growth (Chemurot et al. 2016; and Dolezal et al. 2016). However, the effect of landscape is context‐dependent, which may explain these discrepancies and underscore the need for further site‐specific investigations in future studies, as suggested by Sobkowich et al. (2022). The highly virulent Korean haplotype (K1) of *V. destructor* was detected across all study sites, consistent with its known prevalence in Africa (Begna et al. 2016; Namayanja et al. 2016; Nganso et al. 2017; Traynor et al. 2016). Interestingly, the Amhara honey bees studied herein are surviving mite infestations without miticide treatment, despite mite levels exceeding the economic threshold for acaricide application (3 mites/100 honey bees) (Currie and Gatien 2006), particularly for the highland and lowland bees. Similar natural resilience has been reported in *Varroa*‐surviving honey bees in Kenya (Nganso et al. 2017), South Africa (Mortensen et al. 2016; Strauss et al. 2016), Norway (Oddie et al. 2023), and Brazil (Carneiro et al. 2007).

### 4.2 Grooming Behavior of Ethiopian Honey Bees Against *Varroa* Mites

In this study, the adult grooming rate was significantly influenced by both landscape and season, with no interaction effect, suggesting that these environmental variables influence the expression of this defensive behavioral trait. Notably, lowland and highland honey bees were more aggressive towards *Varroa* mite, particularly during the dry season (Figure 3A). Interestingly, midland honey bees maintained the lowest phoretic mite levels via their effective mite removal, despite inflicting the least damage on *Varroa* mites. This was evidenced by their highest daily natural mite fall per colony and ratio of total fallen mites to adult mite infestation level, especially during the dry season (Figure 4, Table 2). This ratio was similar to that of *Varroa*‐surviving *A. m. scutellata* colonies in Kenya and about six times higher than that of mite‐susceptible *A. mellifera* hybrids of European origin colonies in the USA (Table 2). On one hand, these results suggest that midland honey bees in Ethiopia, which sustain lower adult mite infestation levels, exhibit a more efficient grooming behavior towards *Varroa* mite than their European counterparts. On the other hand, our results also indicate that the percentage of damaged mites might not always be a reliable indicator of adult grooming efficiency within honey bee colonies, as such measure may not fully capture the intensity of grooming activity at the colony level, as previously reported (Nganso et al. 2017). In fact, the observed damage on fallen mites likely reflects those individuals that resist being groomed off, given the gripping adaptations of the parasite itself. *Varroa* mite strategically attaches between the abdominal segments of the honey bees, making detection and removal more challenging (Ramsey et al. 2019). Therefore, mites that sustain damage may represent those individuals with stronger attachment or positioning, leading to higher resistance to grooming attempts by honey bees.

This study also identified, for the first time, two new combinations of damaged categories: hollow in the dorsal shield + damaged leg + damaged gnathosoma, and damaged gnathosoma + damaged shield, in addition to previously reported ones (Corrêa‐Marques et al. 2000; Nganso et al. 2017) (Table 1). Honey bees from the Amhara region most frequently inflicted damaged legs + damaged gnathosoma just like mite‐surviving honey bees in Kenya, unlike the susceptible *A. mellifera* hybrids of European origin in the USA, which damage legs more frequently (Nganso et al. 2017). Overall, this result suggests that the Amhara's honey bee populations displayed high levels of aggression towards *Varroa* mites, with their grooming behavior ranging from 28.0% to 41.9% during the wet season and from 15.7% to 33.8% during the dry season. However, honey bees from both the central highlands and the Tigray region of Ethiopia tend to damage the legs of mites more frequently (Gebremedhn et al. 2019; Gela et al. 2023). This discrepancy may be linked to local adaptation to specific environmental conditions, which is believed to contribute to the morphological differentiation observed among Ethiopian honey bee populations, which belong to the same subspecies, *A. m*. *simensis* (Meixner et al. 2011; Hailu et al. 2020; Wanore et al. 2025).

We found no significant relationship between mite infestation levels and either adult grooming behavior or daily mite fall per colony across seasons, suggesting that grooming behavior may represent a tolerance mechanism employed by Ethiopian honey bees to counteract mites' attack. Moreover, adult mite infestation levels and the ratio of naturally fallen mites to infestation levels during the dry season were negatively correlated, suggesting that increased grooming efforts may occur under high *Varroa* parasitism.

### 4.3 Hygienic Behavior of Ethiopian Honey Bees Against *Varroa* Mites

Our results showed that the hygienic behavior of Ethiopian honey bees from the Amhara region was significantly influenced by landscape and the time interval of dead brood removal, but not by their interaction. During the 24 h interval, lowland and midland honey bees were more hygienic than highland honey bees (Figure 5), suggesting once again that landscape‐related factors may play a role in shaping adult hygienic responses against *Varroa* infestation.

The average brood removal rates after 24 and 48 h exceeded 79.9% and 98.8%, respectively, indicating high general sanitary behavior across all sites. This result corroborates previous reports in other parts of the country (Gela et al. 2023; Gebremedhn et al. 2019; Alemu et al. 2014). Contrary to the report from the central highlands and Tigray region (Gela et al. 2023; Gebremedhn et al. 2019), no correlation was observed between mite infestation and hygienic behavior during either season. Disparities may result from methodological variances across studies or environmental effects.

In general, the mechanisms of tolerance (minimizing harm) and resistance (lowering the reproductive fitness) in mite‐surviving honey bee populations are intricate and influenced by both environmental and genetic factors (Mondet et al. 2020; Rosenkranz et al. 2010). African honey bee populations employ a variety of tactics to survive mite infestations, such as suppression of mite reproduction via high *Varroa‐*specific hygienic behavior (Cheruiyot et al. 2018) or high brood cell recapping rate (Martin et al. 2019), grooming and hygienic behaviors (Gebremedhn et al. 2019; Gela et al. 2023; Muli et al. 2014; Nganso et al. 2017). Additionally, behavioral characteristics like swarming and absconding, highly expressed by African honey bees compared to their European counterparts (Hepburn and Radloff 1988), interrupt mite life cycles by periodically disrupting continuous brood rearing inside the colonies (Nganso et al. 2017, 2024). On the basis of this evidence, it appears that all the mechanisms Ethiopian honey bees employ to counteract mites' attacks within their hives are not yet fully understood, warranting further research.

## 5 Conclusions

In the present study, *A. m. simensis* from the Amhara region survived *Varroa* mite infestation without the use of miticide, even though mite infestation levels passed the recommended threshold for acaricide treatment. Adult mite infestation levels were significantly influenced by both season and landscape. Infestations were less pronounced during the dry season in midland colonies compared to colonies in the lowland and highland areas. Adult mite infestation levels also varied substantially across honey bee subspecies during the dry season, with *A. m. simensis* colonies from Ethiopia showing lower mite loads than those of *A. m. scutellata* in Kenya and *A. mellifera* hybrids of European origin in the USA. Our study further highlights that the grooming behavior of these honey bees from the Amhara region against *Varroa* mites was influenced by both landscape and season, with midland honey bees exhibiting the highest daily mite fall per colony and ratio of total natural fallen mite/adult mite infestation rate, especially during the dry season, which was similar to that of the mite‐surviving *A. m. scutellata* colonies in Kenya. Likewise, hygienic behavior was significantly influenced by landscape, with midland and lowland honey bees exhibiting higher brood removal rates than highland bees. These findings suggest that environmental factors play a crucial role in shaping adult hygienic and grooming responses against *Varroa*. However, the lack of significant correlations between adult mite infestation levels and either grooming or hygienic behavior suggests that these behavioral traits function as tolerant mechanisms, limiting the detrimental effects of the mite on their honey bee host in Amhara. Other resistance behavioral mechanisms, such as suppression of mite reproduction potential, are likely involved in reducing mite loads within Amhara's honey bee colonies. Another possibility is that the relationship between these behaviors and mite levels may not be linear but rather could follow a threshold pattern. Given that the effect of landscape appears context‐specific, further site‐specific investigations across other lowland, midland, and highland areas in the future are recommended to better understand how environmental variation influences resistance and tolerance mechanisms in *A. m. simensis*.

## Author Contributions

**Walellign W. Wanore:** data curation (lead), formal analysis (lead), investigation (lead), visualization (lead), writing – review and editing (lead). **Christian W. W. Pirk:** conceptualization (supporting), data curation (supporting), formal analysis (supporting), methodology (supporting), supervision (equal), writing – review and editing (equal). **Abdullahi A. Yusuf:** conceptualization (supporting), data curation (supporting), formal analysis (supporting), methodology (equal), supervision (equal), writing – review and editing (equal). **Workneh Ayalew:** funding acquisition (lead), project administration (lead), writing – review and editing (supporting). **Beatrice T. Nganso:** conceptualization (lead), data curation (supporting), formal analysis (supporting), funding acquisition (equal), methodology (lead), supervision (equal), visualization (lead), writing – review and editing (lead).

## Funding

Data collection in Ethiopia and analysis at *icipe* was funded by the Mastercard Foundation through the More Young Entrepreneurs in Silk and Honey (MOYESH) Programme. The authors acknowledge the financial support for this research by the following organizations and agencies: the Swedish International Development Cooperation Agency (Sida); the Swiss Agency for Development and Cooperation (SDC); the Australian Centre for International Agricultural Research (ACIAR); the Government of Norway; the German Federal Ministry for Economic Cooperation and Development (BMZ); and the Government of the Republic of Kenya. The views expressed herein do not necessarily reflect the official opinion of the donors. Walellign W. Wanore acknowledges the German Academic Exchange Service In‐region Scholarship (DAAD) and the University of Pretoria in South Africa for funding his PhD research work and studies. Financial support was as well provided to A. Yusuf and C. Pirk through the Competitive Program for Rated Research by the National Research Foundation of South Africa.

## Conflicts of Interest

The authors declare no conflicts of interest.

## Supporting information


**Data S1:** ece372660‐sup‐0001‐DataS1.xlsx.

**Data S2:** ece372660‐sup‐0002‐DataS2.xlsx.

**Data S3:** ece372660‐sup‐0003‐DataS3.xlsx.

**Data S4:** ece372660‐sup‐0004‐DataS4.xlsx.

**Data S5:** ece372660‐sup‐0005‐DataS5.xlsx.

**Table S1–S2:** ece372660‐sup‐0006‐TableS1‐S2.docx.

## Data Availability

All relevant data is within the manuscript and its [Link], [Link], [Link], [Link], [Link] files.

## References

[ece372660-bib-0002] Alemu, T. , G. Legesse , and Z. Ararso . 2014. “Performance Evaluation of Honeybee (*Apis mellifera scutellata*) in Guji Zone.” International Journal of Innovation and Applied Studies 9, no. 4: 1987–1993. http://www.ijias.issr‐journals.org/abstract.php?article=IJIAS‐14‐281‐06.

[ece372660-bib-0003] Aronstein, K. A. , E. Saldivar , R. Vega , S. Westmiller , and A. E. Douglas . 2012. “How *Varroa* Parasitism Affects the Immunological and Nutritional Status of the Honey Bee, *Apis mellifera* .” Insects 3, no. 3: 601–615. 10.3390/insects3030601. 26466617 PMC4553578

[ece372660-bib-0004] Begna, D. , A. Gela , T. Negera , and A. Bezabeh . 2016. “Identifying the Species, Effects and Seasonal Dynamics of Honeybee *Varroa* Mites: A Newly Emerging Parasite to Ethiopian Honeybee.” International Journal of Scientific Research in Environmental Science and Toxicology 2, no. 1: 1–4. 10.15226/2572-3162/2/1/00102.

[ece372660-bib-0005] Bienefeld, K. , F. Zautke , D. Pronin , and A. Mazeed . 1999. “Recording the Proportion of Damaged *Varroa jacobsoni* Oud. In the Debris of Honey Bee Colonies (*Apis mellifera*).” Apidologie 30: 249–256. 10.1051/apido:19990401.

[ece372660-bib-0006] Boecking, O. , and W. Ritter . 1993. “Grooming and Removal Behaviour of *Apis mellifera* Intermissa in Tunisia Against *Varroa jacobsoni* .” Journal of Apicultural Research 24, no. 6: 127–134. 10.1080/00033799300200371.

[ece372660-bib-0007] Bruckner, S. , N. Steinhauer , J. Engelsma , et al. 2020. 2019–2020 Honey Bee Colony Losses in the United States: Preliminary Results.

[ece372660-bib-0008] Carneiro, F. E. , R. R. Torres , R. Strapazzon , et al. 2007. “Changes in the Reproductive Ability of the Mite *Varroa destructor* (Anderson Trueman) in Africanized Honey Bees (*Apis mellifera* L.) (Hymenoptera: Apidae) Colonies in Southern Brazil.” Neotropical Entomology 36, no. 6: 949–952. 10.1590/S1519-566X2007000600018. 18246271

[ece372660-bib-0009] Ceccotti, M. , C. Miotti , A. Pacini , M. Signorini , and A. Giacobino . 2022. “ *Varroa destructor* and *Nosema sp* Seasonal Dynamics in *Apis mellifera* Colonies From Temperate Climate in Argentina.” Revista Veterinaria 33, no. 1: 87–93. 10.30972/vet.3315889.

[ece372660-bib-0010] Chemurot, M. , A. M. Akol , C. Masembe , L. de Smet , T. Descamps , and D. C. de Graaf . 2016. “Factors Influencing the Prevalence and Infestation Levels of *Varroa destructor* in Honeybee Colonies in Two Highland Agro‐Ecological Zones of Uganda.” Experimental and Applied Acarology 68: 497–508. 10.1007/s10493-016-0013-x. 26801158

[ece372660-bib-0011] Cheruiyot, S. K. , H. M. G. Lattorff , R. Kahuthia‐Gathu , J. P. Mbugi , and E. Muli . 2018. “ *Varroa*‐Specific Hygienic Behavior of *Apis mellifera scutellata* in Kenya.” Apidologie 49, no. 4: 439–449. 10.1007/s13592-018-0570-6.

[ece372660-bib-0012] Corrêa‐Marques, M. H. , M. R. C. Issa , and D. De Jong . 2000. “Classification and Quantification of Damaged *Varroa jacobsoni* Found in the Debris of Honey Bee Colonies as Criteria for Selection?” American Bee Journal 140, no. 10: 820–824.

[ece372660-bib-0013] CSA (Central Statistical Agency) . 2021. “Report on Land Utilization by Private Peasant Holdings in 2020/2021 Meher Cropping Season.” Statistical Bulletin, 21.

[ece372660-bib-0014] Currie, R. W. , and P. Gatien . 2006. “Timing Acaricide Treatments to Prevent *Varroa destructor* (Acari: Varroidae) From Causing Economic Damage to Honey Bee Colonies.” Entomological Society of Canada 138, no. 2006: 238–252.

[ece372660-bib-0015] Dafar, A. , and M. Turi . 2019. “Analysis Marketing Behavior of Honey in Oromia Regional State.” Journal of Marketing and Consumer Research 57, no. 2017: 1–13. 10.7176/JMCR.

[ece372660-bib-0017] de Souza, F. S. , M. H. Allsopp , and S. J. Martin . 2021. “Deformed Wing Virus Prevalence and Load in Honey Bees in South Africa.” Archives of Virology 166, no. 1: 237–241. 10.1007/s00705-020-04863-5. 33136209 PMC7815608

[ece372660-bib-0018] Dietemann, V. , F. Nazzi , S. J. Martin , et al. 2013. “Standard Methods for *Varroa* Research.” Journal of Apicultural Research 52, no. 1: 37–41. 10.3896/IBRA.1.52.1.09.

[ece372660-bib-0019] Dolezal, A. G. , J. Carrillo‐Tripp , W. Allen Miller , B. C. Bonning , and A. L. Toth . 2016. “Intensively Cultivated Landscape and *Varroa* Mite Infestation Are Associated With Reduced Honey Bee Nutritional State.” PLoS One 11, no. 4: 1–13. 10.1371/journal.pone.0153531. PMC4829173 27070422

[ece372660-bib-0020] Francis, R. M. , S. L. Nielsen , and P. Kryger . 2013. “ *Varroa*‐Virus Interaction in Collapsing Honey Bee Colonies.” PLoS One 8, no. 3: e57540. 10.1371/journal.pone.0057540. 23526946 PMC3602523

[ece372660-bib-0021] Gebremedhn, H. , B. Amssalu , L. De Smet , and D. C. de Graaf . 2019. “Factors Restraining the Population Growth of *Varroa Destructor* in Ethiopian Honey Bees (*Apis Mellifera Simensis*).” PLoS One 14, no. 9: 1–16. 10.1371/journal.pone.0223236. PMC6762127 31557264

[ece372660-bib-0022] Gela, A. , A. Gebresilassie , Y. Woldehawariat , A. Atikem , Z. Ararso , and A. Bezabeh . 2023. “Defensive Behaviors of the Central Highland Honeybees, *Apis mellifera bandasii* Against *Varroa destructor* in Ethiopia.” Bee Studies 15, no. 2: 61–71.

[ece372660-bib-0023] Gray, A. , N. Adjlane , A. Arab , et al. 2023. “Honey Bee Colony Loss Rates in 37 Countries Using the COLOSS Survey for Winter 2019–2020: The Combined Effects of Operation Size, Migration and Queen Replacement.” Journal of Apicultural Research 62, no. 2: 204–210.

[ece372660-bib-0024] Grozinger, C. M. , and M. L. Flenniken . 2019. “Bee Viruses: Ecology, Pathogenicity, and Impacts.” Annual Review of Entomology 64: 205–226. 10.1146/annurev-ento-011118-111942. 30629896

[ece372660-bib-0025] Hailu, T. G. , P. D'alvise , A. Tofilski , et al. 2020. “Insights Into Ethiopian Honey Bee Diversity Based on Wing Geomorphometric and Mitochondrial DNA Analyses.” Apidologie 51: 1182–1198. 10.1007/s13592-020-00796-9.

[ece372660-bib-0026] Hall, T. A. 1999. “BioEdit: A User‐Friendly Biological Sequence Alignment Editor and Analysis Program for Windows 95/98/NT.” Nucleic Acids Symposium Series 41: 95–98.

[ece372660-bib-0027] Hepburn, H. R. , and S. E. Radloff . 1988. Honey Bees of Africa. Springer Verlag.

[ece372660-bib-0028] Hristov, P. , R. Shumkova , N. Palova , and B. Neov . 2020. “Factors Associated With Honey Bee Colony Losses: A Mini‐Review.” Veterinary Sciences 7, no. 4: 166. 33143134 10.3390/vetsci7040166 PMC7712510

[ece372660-bib-0029] Jack, C. J. , I. de Bem Oliveira , C. B. Kimmel , and J. D. Ellis . 2023. “Seasonal Differences in *Varroa destructor* Population Growth in Western Honey Bee ( *Apis mellifera* ) Colonies.” Frontiers in Ecology and Evolution 11: 1102457. 10.3389/fevo.2023.1102457.

[ece372660-bib-0030] Lee, K. V. , N. Steinhauer , K. Rennich , et al. 2015. “A National Survey of Managed Honey Bee 2013–2014 Annual Colony Losses in the USA.” Apidologie 46, no. 3: 292–305. 10.1007/s13592-015-0356-z.

[ece372660-bib-0031] Lenth, R. 2023. “Emmeans: Estimated Marginal Means, Aka Least‐Squares Means.” R Package Version 1.8.5.

[ece372660-bib-0032] Locke, B. 2016. “Natural *Varroa* Mite‐Surviving *Apis mellifera* Honeybee Populations.” Apidologie 47, no. 3: 467–482. 10.1007/s13592-015-0412-8.

[ece372660-bib-0033] Locke, B. , and I. Fries . 2011. “Characteristics of Honey Bee Colonies ( *Apis mellifera* ) in Sweden Surviving *Varroa destructor* Infestation.” Apidologie 42, no. 4: 533–542. 10.1007/s13592-011-0029-5.

[ece372660-bib-0035] Martin, S. J. , G. P. Hawkins , L. E. Brettell , N. Reece , M. E. Correia‐Oliveira , and M. H. Allsopp . 2019. “ *Varroa destructor* Reproduction and Cell Re‐Capping in Mite‐Resistant *Apis mellifera* Populations.” Apidologie 51, no. 3: 369–381. 10.1007/s13592-019-00721-9.

[ece372660-bib-0036] Meixner, M. D. , M. A. Leta , N. Koeniger , and S. Fuchs . 2011. “The Honey Bees of Ethiopia Represent a New Subspecies of * Apis mellifera—Apis mellifera simensis * N. Ssp.” Apidologie 42: 425–437. 10.1007/s13592-011-0007-y.

[ece372660-bib-0037] Midekisa, A. , B. Beyene , A. Mihretie , E. Bayabil , and M. C. Wimberly . 2015. “Seasonal Associations of Climatic Drivers and Malaria in the Highlands of Ethiopia.” Parasites and Vectors 8, no. 1: 339. 10.1186/s13071-015-0954-7. 26104276 PMC4488986

[ece372660-bib-0038] Mondet, F. , A. Beaurepaire , A. McAfee , et al. 2020. “Honey Bee Survival Mechanisms Against the Parasite *Varroa destructor*: A Systematic Review of Phenotypic and Genomic Research Efforts.” International Journal for Parasitology 50, no. 2020: 433–447. 10.1016/j.ijpara.2020.03.005. 32380096

[ece372660-bib-0039] Mortensen, A. N. , D. R. Schmehl , M. Allsopp , et al. 2016. “Differences in *Varroa destructor* Infestation Rates of Two Indigenous Subspecies of *Apis mellifera* in the Republic of South Africa.” Experimental and Applied Acarology 68, no. 4: 509–515. 10.1007/s10493-015-9999-8. 26704261

[ece372660-bib-0040] Muli, E. , H. Patch , M. Frazier , et al. 2014. “Evaluation of the Distribution and Impacts of Parasites, Pathogens, and Pesticides on Honey Bee ( *Apis mellifera* ) Populations in East Africa.” PLoS One 9, no. 4: e94459. 10.1371/journal.pone.0094459. 24740399 PMC3989218

[ece372660-bib-0041] Namayanja, D. , A. M. Akol , and D. R. Kugonza . 2016. “Prevalence of *Varroa* Mite Infestations Among Honey Bee Colonies in Uganda.” *Fifth African Higher Education Week and RUFORUM Biennial Conference 2016*, “Linking Agricultural Universities With Civil Society, the Private Sector, Governments and Other Stakeholders in Support of Agricultural Development in Africa”, Cape Town, South Africa, 14(14), 459–465.

[ece372660-bib-0042] Nearman, A. , C. L. Crawford , M. M. Guarna , et al. 2025. “Insights From US Beekeeper Triage Surveys Following Unusually High Honey Bee Colony Losses 2024–2025.” Science of the Total Environment 1003: 180650. 41061511 10.1016/j.scitotenv.2025.180650

[ece372660-bib-0043] Nganso, B. T. , W. Ayalew , A. J. Wubie , et al. 2025. “Honey Bee Colony Losses and Causes During the Active Beekeeping Season 2022/2023 in Nine Sub‐Saharan African Countries.” PLoS One 20, no. 5: e0322489. 10.1371/journal.pone.0322489. 40388478 PMC12088056

[ece372660-bib-0044] Nganso, B. T. , A. T. Fombong , A. A. Yusuf , C. W. W. Pirk , C. Stuhl , and B. Torto . 2017. “Hygienic and Grooming Behaviors in African and European Honey Bees—New Damage Categories in *Varroa destructor* .” PLoS One 12, no. 6: e0179329. 10.1371/journal.pone.0179329. 28622341 PMC5473549

[ece372660-bib-0045] Nganso, B. T. , A. T. Fombong , A. A. Yusuf , C. W. W. Pirk , C. Stuhl , and B. Torto . 2018. “Low Fertility, Fecundity and Numbers of Mated Female Offspring Explain the Lower Reproductive Success of the Parasitic Mite *Varroa destructor* in African Honey Bees.” Parasitology 145, no. 12: 1633–1639. 10.1017/S0031182018000616. 29661259

[ece372660-bib-0046] Nganso, B. T. , V. Soroker , A. F. Osabutey , et al. 2024. “Best Practices for Colony Management: A Neglected Aspect for Improving Honey Bee Colony Health and Productivity in Africa.” Journal of Apicultural Research 63, no. 3: 438–455. 10.1080/00218839.2024.2308418.

[ece372660-bib-0047] O'Connell, D. P. , K. Healy , J. Wilton , C. Botías , and J. C. Jones . 2025. “A Systematic Meta‐Analysis of the Efficacy of Treatments for a Global Honey Bee Pathogen‐The Varroa Mite.” Science of the Total Environment 963: 178228. 10.1016/j.scitotenv.2024.178228. 39837751

[ece372660-bib-0048] Oddie, M. A. Y. , S. Lanz , B. Dahle , O. Yañez , and P. Neumann . 2023. “Virus Infections in Honeybee Colonies Naturally Surviving Ectoparasitic Mite Vectors.” PLoS One 18, no. 12: e0289883. 10.1371/journal.pone.0289883. 38100484 PMC10723705

[ece372660-bib-0049] Pirk, C. W. W. , H. Human , R. M. Crewe , and D. vanEngelsdorp . 2014. “A Survey of Managed Honey Bee Colony Losses in the Republic of South Africa–2009 to 2011.” Journal of Apicultural Research 53, no. 1: 35–42. 10.3896/ibra.1.53.1.03.

[ece372660-bib-0051] R Core Team . 2023. R: A Language and Environment for Statistical Computing (4.3.2). R Foundation for Statistical Computing.

[ece372660-bib-0052] Ramsey, S. D. , R. Ochoa , G. Bauchan , et al. 2019. “ *Varroa destructor* Feeds Primarily on Honey Bee Fat Body Tissue and Not Hemolymph.” Proceedings of the National Academy of Sciences of the United States of America 116, no. 5: 1792–1801. 10.1073/pnas.1818371116. 30647116 PMC6358713

[ece372660-bib-0053] Rath, W. 1999. “Co‐Adaptation of *Apis cerana* Fabr. and *Varroa jacobsoni* Oud.” Apidologie 30, no. 2–3: 97–110. 10.1111/j.1742-6723.2011.01433.x.

[ece372660-bib-0057] Rosenkranz, P. , P. Aumeier , and B. Ziegelmann . 2010. “Biology and Control of *Varroa destructor* .” Journal of Invertebrate Pathology 103: S96–S119. 10.1016/j.jip.2009.07.016. 19909970

[ece372660-bib-0059] Sobkowich, K. E. , O. Berke , T. M. Bernardo , D. L. Pearl , and P. Kozak . 2022. “Spatial Analysis of *Varroa destructor* and the Relationship With Surrounding Landscape Types in Southern Ontario.” Frontiers in Ecology and Evolution 10: 1027297. 10.3389/fevo.2022.1027297.

[ece372660-bib-0060] Soroker, V. , A. Hetzroni , B. Yakobson , et al. 2011. “Evaluation of Colony Losses in Israel in Relation to the Incidence of Pathogens and Pests.” Apidologie 42, no. 2: 192–199. 10.1051/apido/2010047.

[ece372660-bib-0061] Spivak, M. , and R. G. Danka . 2021. “Perspectives on Hygienic Behavior in *Apis mellifera* and Other Social Insects.” Apidologie 52, no. 1: 1–16. 10.1007/s13592-020-00784-z.

[ece372660-bib-0062] Strauss, U. , V. Dietemann , H. Human , R. M. Crewe , and C. W. W. Pirk . 2016. “Resistance Rather Than Tolerance Explains Survival of Savannah Honey Bees (*Apis mellifera scutellata*) to Infestation by the Parasitic Mite *Varroa destructor* .” Parasitology 143, no. 3: 374–387. 10.1017/S0031182015001754. 26690678

[ece372660-bib-0063] Strauss, U. , H. Human , L. Gauthier , R. M. Crewe , V. Dietemann , and C. W. Pirk . 2013. “Seasonal Prevalence of Pathogens and Parasites in the Savannah Honeybee (*Apis mellifera scutellata*).” Journal of Invertebrate Pathology 114, no. 1: 45–52. 23702244 10.1016/j.jip.2013.05.003

[ece372660-bib-0066] Traynor, K. S. , F. Mondet , J. R. de Miranda , et al. 2020. “ *Varroa destructor* : A Complex Parasite, Crippling Honey Bees Worldwide.” Trends in Parasitology 36, no. 7: 592–606. 10.1016/j.pt.2020.04.004. 32456963

[ece372660-bib-0067] Traynor, K. S. , K. Rennich , E. Forsgren , et al. 2016. “Multiyear Survey Targeting Disease Incidence in US Honey Bees.” Apidologie 47: 325–347. 10.1007/s13592-016-0431-0.

[ece372660-bib-0068] Wanore, W. W. , C. W. Pirk , A. A. Yusuf , et al. 2025. “Honey Bees of Ethiopia: Their Lineages and Subspecies Based on Morphometrics, Mitochondrial DNA, and Mandibular Gland Pheromone Analyses.” PLoS One 20, no. 11: e0335551. 10.1371/journal.pone.0335551. 41202028 PMC12594397

[ece372660-bib-0069] Wilkinson, D. , and G. C. Smith . 2002. “A Model of the Mite Parasite, *Varroa destructor*, on Honey Bees (*Apis mellifera*) to Investigate Parameters Important to Mite Population Growth.” Ecological Modelling 148: 263–275. www.elsevier.com/locate/ecolmodel.

