# Supplementary material for: Fending for Thyself: Honey Bees From Ethiopia Inflict Physical Damage on Varroa destructor
Source: Ecol Evol. 2025 Dec 16;15(12):e72660. doi: 10.1002/ece3.72660 (PMC12706522; doi:10.1002/ece3.72660)
Supplement: Supplementary file 6 — Table S1–S2: ece372660‐sup‐0006‐TableS1‐S2.docx. [file ECE3-15-e72660-s004.docx]

**Supplementary information**

**Table S1**. The average relative humidity, maximum and minimum temperatures of Awi, East Gojjam, and South Gondar provinces in the Amhara region during the wet (November – December) and the dry (January – February) seasons. The data was extracted from Google Earth Engine (GEE); temperature data was sourced from https://developers.google.com/earth-engine/datasets/catalog/ECMWF_ERA5_LAND_DAILY_AGGR, while relative humidity data was sourced from https://developers.google.com/earth-engine/datasets/catalog/ECMWF_ERA5_LAND_HOURLY.

| Provinces | Season | Average relative humidity (%) | Average temperature (^0^C) | |
| --- | --- | --- | --- | --- |
|  |  |  | Minimum | Maximum |
| Awi | Wet | 86.49 | 28.60 | 29.68 |
|  | Dry | 77.58 | 28.72 | 30.00 |
| East Gojjam | Wet | 86.37 | 28.22 | 29.27 |
|  | Dry | 78.73 | 28.18 | 29.52 |
| South Gondar | Wet | 82.76 | 28.49 | 29.58 |
|  | Dry | 78.49 | 28.48 | 29.72 |

**Table S2.** The primer sequences and their annealing temperatures

| **Fragment** | **Primer name** | **Primer sequences (5’-3’)** | **Size (bp)** | **Ta (◦C)** |
| --- | --- | --- | --- | --- |
| *cox1* | 10KbCOIF1  6,5KbCOIR | CTT GTA ATC ATA AGG ATA TTG GAAC  AAT ACC AGT GGG AAC CGC | 929 | 52 |
| *atp6-cox3* | 6KbATP6F  16KbCOIIIR | GAC ATA TAT CAG TAA CAA TGAG  GAC TCC AAG TAA TAG TAA AACC | 818 | 52 |
